# A phenotypic and molecular investigation of biofilm formation in clinical samples of *Pseudomonas aeruginosa*

**DOI:** 10.22099/mbrc.2021.41708.1673

**Published:** 2021-12

**Authors:** Leila Dolatshah, Mohammad Tabatabaei

**Affiliations:** Department of Pathobiology, School of Veterinary Medicine, Shiraz University, Shiraz, Iran

**Keywords:** Pseudomonas aeruginosa, Biofilm, Quorum sensing, Fimbrial, cupA, pslA

## Abstract

*Pseudomonas aeruginosa* is identified as a versatile opportunistic microorganism with metabolic diversity contributing to a wide range of health burdens, especially in immunocompromised patients. This bacterium is the cause of 10 to 20% of nosocomial infections. In this study, we evaluated the phenotypic characterizations of biofilm formation in *P. aeruginosa* clinical isolates using micro-titer plate assay. Indeed, we estimated the prevalence of QS (*rhlI*, *rhlR*, *rhlAB*, *lasB*, *lasI*, *lasR, aprA*) and virulence genes (*pslA* and *cupA*) by PCR. The results showed that among 69% of the isolates forming biofilm, 9% were strong biofilm producers, whereas 13% and 47% of isolates produced moderate and low amounts of biofilm, respectively. All isolates possessed *cupA *and seven QS genes (*rhlI*, *rhlR*, *rhlAB*, *lasB*,  *lasI*, *lasR*, *aprA*), while 92% of the isolates possessed the *pslA* gene. Identification of these genes and their association with biofilm formation can be advantageous in adopting therapeutic methods.

## INTRODUCTION

*Pseudomonas aeruginosa* is a rod-shaped, gram-negative, opportunistic versatile pathogen bacterium [1] leading to acute as well chronic infections in intensive care unit (ICU), immunocompromised, and cystic fibrosis patients [2,3]. Up to 10-20% of nosocomial infections are notably associated with *P. aeruginosa *pathogenesis. The World Health Organization (WHO) has classified this organism as the first antibiotic-resistance human pathogen making it necessary to develop novel antibacterial agents [4-7]. 

Microbial communities are known as biofilms commonly exist in environmental and clinical settings [8]. They cause antibiotic resistance and help bacteria to evade the host immune system [9,10]. In this regard, *P. aeruginosa* can produce biofilm in the respiratory tract or pulmonary tissue of cystic fibrosis patients (CF) and on abiotic surfaces such as contact lenses and catheters [11,12]. Exopolysaccharides (EPSs) are a major constituent of microbial biofilms [13]. At least three EPSs including alginate, Pel, and Psl have been identified as associated with biofilm formation *in P. aeruginosa* [15]. In this context, Ma et al., (2006) demonstrated that Psl polysaccharide plays a significant role in the attachment of *P. aeruginosa* colony-biofilms to both abiotic and biotic surfaces at the primary phases. It as well improves the maintenance of biofilm structure after adherence. It is also counted as that Psl serves as a scaffold for other biofilm components preserving the natural structure of biofilm [14].

The *cupA *gene cluster, another key player in the pathogenesis of *P. aeruginosa*, is more responsible for biofilm development during the early stages compared to type IV pili [16]. The expression of many virulence genes, including biofilm-associated factors in *P. aeruginosa *is regulated by Quorum sensing (QS) network [17]. QS is a complicated microbial cell-cell mechanism entailing in the production and maintenance of biofilm. Two QS systems, the *las* and *rhl *systems have been identified in *P. aeruginosa *so far. In this context, the LasI system controls the formation of the homoserine lactone (3-oxo-C12) signal molecule which plays a key role in forming biofilms [17,18]. It reacts with the LasR activator and in addition to positive feedback on itself, triggers several other virulence genes including *lasB*, *lasA*, *aprA*, and *toxA *[19].

This study aimed to evaluate the phenotypic biofilm formation and prevalence of aforementioned QS and virulence genes in the isolates cultured from clinical cases of *P*. *aeruginosa* infection.

## MATERIALS AND METHODS

**Bacterial strain**: The bacteria used in this study were isolated from various clinical specimens (urine, skin, sputum, body fluid, blood, wound, central vein blood). They included PAO1, and 100 strains of *P. aeuginosa *stored in a bacterial collection of the School of Veterinary Medicine, Shiraz University.

**Biofilm assay: **The biofilm was developed on a 96-well polystyrene micro-titre plate according to Christensen et al., with some changes [20]. Concisely, biofilm bacteria were grown in trypticase soy broth (TSB) medium (MERK Germany) enriched with 1% glucose (BDH England). After incubation at 37°C for 24 hours, the bacterial suspensions were diluted 1/100 with sterile fresh TSB containing 1% glucose. A 200 µl of diluted microbial suspension was poured into the 96-well polystyrene Plates (SPL Korea) in triplicate. Negative controls only consisting sterile TSB medium. Three wells were used for each sample. Afterward, the plates were covered and incubated at 37°C for 24 hours. Subsequently, the solution content of the wells was aspirated and the wells were washed three times by addition 200 µl sterile phosphate-buffered saline (PBS). The formed biofilms were fixed with absolute methanol (Merck Germany). After 15 minutes the plates were rinsed off with PBS and air-dried. The wells were stained with 200 µl of 1% crystal violet solution (Merck Germany). Excess stain was removed using sterile distilled water. Finally, stained biofilms dissolved in 33% (v/v) glacial acetic acid (Merck Germany). The OD value (ODw) of each well was provided at 570 nm using an ELISA reader (Biotek USA). All strains were categorized as represented by Stepanovic et al. [21]. The cut-off OD value (ODc) for each sample was described as three standard deviations above the mean OD of the negative control. The strains were introduced into four following groups according to the ODw: non-biofilm formation (0) (ODw ≤ ODc); weakly biofilm formation (+) (ODc<ODw≤2xODc); moderately biofilm formation (++) (2xODc < ODw ≤ 4xODc); and strongly biofilm formation (+++) (4xODc < ODw). 

**PCR for detection of biofilm-related and QS genes: **The bacterial isolates were evaluated for seven genes including Qs genes (*rhlI*, *rhlR*, *rhlAB*, *lasB*, *lasI*, *lasR*, *aprA*) and two other genes (*cupA*, *pslA*) contributing to biofilm formation by PCR. Nine primer pairs were used for polymerization, as previously described (Table 1) [19,22,23]. DNA extraction was performed using the boiling method. The PCR reaction mixture contained10 µl Master mix (1.5 X AMPLICON DENMARK), 0.5 µl of 10 pmol forward and reverse primers concentration, 2.5 µl DNA, and 6.5 µl of nuclease-free water. PCR programs for the detection of different genes are described in Table 2.

**Table 1:** Primers used for detection of the Quorum-Sensing and virulence genes

**Genes**	**Sequence of primers**	**Amplicon Size (bp)**	**References**
***rhlI***	5′-TTC ATC CTC CTT TAG TTC TTC C 3′ 5′-TTC CAG CGA TTC AGA GAG C-3′	155	
***rhlR***	5′-TGC ATT TTA TCG ATC AGG GC-3′ 5′-CAC TTC CTT TTC CAG GAC G-3′	133	
***rhlAB***	5′-TCA TGG AAT TGT CAC AAC CGC-3′ 5′- ATA CGG CAA AAT CAT GGC AAA C-3′	151	
***lasB***	5′-TTC TAC CCG AAG GAC TGA TAC-3′ 5′-AAC ACC CAT GAT CGC AAC-3′	153	
***lasI***	5′-CGT GCT CAA GTG TTC AAG-3′ 5′-TAC AGT CGG AAA AGC CCA G-3′	295	
***lasR***	5′-AAG TGG AAA ATT GGA GTG GAG-3′ 5′-GTA GTT GCC GAC GAC GAT GAA G-3′	130	
***aprA***	5′-ACC CTG TCC TAT TCG TTC C-3′ 5′-GAT TGC AGC GAC AAC TTG G-3′	140	
***cupA***	5′-CTA CCG CTA TTC CAC CGA AG-3′ 5′-AGG AGC CGG AAA GAT AGA GG-3′	172	
***pslA***	5′-CAC TGG ACG TCT ACT CCG ACG ATA T-3′ 5′-GTT TCT TGA TCT TGT GCA GGG TGT C-3′	1119	

**Table 2:** PCR programs for detection of different genes

**Genes/Steps**	**QS**	***cupA***	***pslA***
Initial denaturation	94°C/ 5min	94°C/ 5min	95°C/ 5min
Denaturation	94°C/ 1min	95°C/ 40 sec	94°C/ 30 sec
Annealing	56°C/ 1min	59°C/ 45 sec	55°C/ 30 sec
Extension	72°C/ 1 min	72°C/ 1 min	72°C/ 1 min
Final extension	72°C/ 8min	72°C/ 7 min	72°C/ 10 min
Cycle	32	40	30

## RESULTS AND DISCUSSION

**Figure 1:**
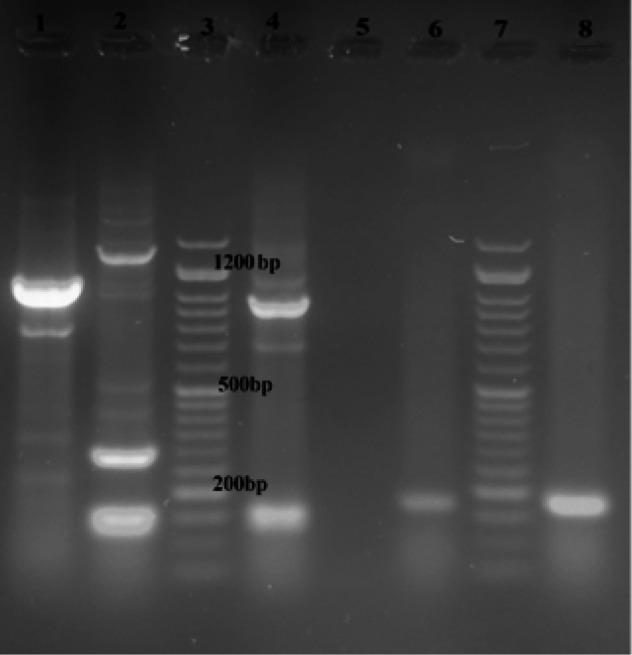
Agaros gel electrophoresis, Lane 1. *pslA* gene (1119bp), Lane 2. *lasB*, *lasI*, and *lasR *genes (153, 295 and 130bp), Lane 3. 50bp DNA ladder, Lane 4. *rhlI* and *rhlR* genes (133 and 155bp), Lane 5. Negetive control, Lanes 6 and 8 *cupA *gene (172bp).

**Figure 2:**
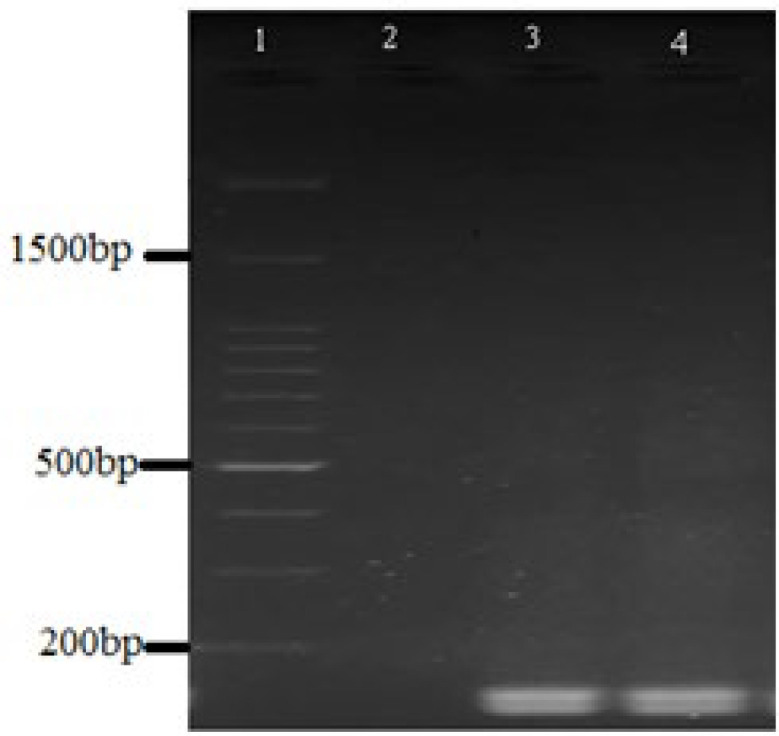
Agaros gel electrophoresis, Lane 1. 100bp DNA ladder, Lane 2. Negative control, Lanes 3 and 4. *aprA *and *rhlB *genes (140 and 151bp).

In the present investigation, 100 clinical samples of *P. aeruginosa* were assessed for biofilm formation and the presence of QS, fimbrial *cupA *and *pslA *genes. In total, 69% of the isolates formed biofilm of which 9% shaped strong biofilm; 13% generated moderate biofilm and 47% formed weak biofilm. All of the isolates (100%) possessed seven QS genes (*rhlI*, *rhlR*, *rhlAB*, *lasB*, *lasI*, *lasR, aprA*) and *cupA* gene, while 92% (92/100) of the isolates possessed *pslA* gene (Fig. 1 and 2).

The quantitative micro-titer plate assay, which is an efficacious method for biofilm detection has been used. Similar to our results, Pereze et al., [24] reported that 68% of the isolates formed biofilm. In other studies by Ghadaksaz et al., [25], and Lima et al. [26] 50.9 and 58.1% of isolates formed biofilm, respectively. Heidari and Eftekhar [27] showed that 43% of the isolates formed biofilm, of which 66.7% were strong and 33.3% were weak producers. The results of these studies were lower than our research. In contrast, Banar et al. [28], showed that more than 96% of isolates causing burn wound infection produced biofilm, of which 30.9% formed strong biofilm, 47.3% formed moderate biofilm and 21.8% formed weak biofilm. Likewise, in another research, Kamali et al. [29], indicated that among 83.75% of the isolates formed biofilm, 16.25% produced strong biofilm; 33.75% produced moderate biofilm; and 33.75% produced weak biofilm, while 16.25% of isolates did not produce any biofilm. Lima et al., [30], indicated that while 25% of isolates were non-adherent, 40% of them were weakly adherent, 25% were moderately adherent, and 10% were firmly adherent. Furthermore, in another study conducted by Lima et al., [31], among 77.5% of isolates produced biofilm, 42.5% were weakly adherent, 27.5% were moderately adherent and 7.5% were firmly adherent. Collectively, in most of these studies, the number of isolates forming weak biofilm was higher than other isolates, confirming our findings.

Biofilm formation is induced and regulated by numerous genes and environmental factors [32]. QS controls about 10% of genes in *P. aeruginosa *[33]. Therefore, the QS system is a potential target for developing novel therapies against *P. aeruginosa *infection. In this study, the genotypic analysis showed that all the isolates had seven QS genes mentioned earlier. Lima et al. [26] showed that four genes including *lasI*, *lasR*, *rhlI*, and *rhlR* were present in the isolates. Additionally, in another study by Lima et al., [31], 100 % of strains were positive for the *lasR*, *rhlI* and *rhlR* genes, and 97.5 % of them were positive for the *lasI *gene. Perez et al., [34], indicated that 90.1 % of isolates possessed *lasI*, *lasR*, *rhlI*, and *rhlR* genes. Moreover, Kadhim and Ali [35], reported that 81.6% of the isolates contained QS genes, among which the frequency of *lasR*, *lasI*, *rhlR,* and *rhlI* genes were 5, 78.3, 65, and 43.3%, respectively.

In the present study, the genes needed for biofilm organization were found in all isolates. However, 31% of the samples were not able to develop biofilms. This may be the result of some point mutations that occurred in the QS genes [36,37]. Another possibility is that the presence of several strains of *P. aeruginosa* at the site of the infection may lead to defective expression of QS genes [37].

All the studied isolates possessed the *cupA* gene. Similarly, Shafiei et al. [22] analyzed four clinical isolates and two standard strains of *P. aeruginosa *and showed that the *cupA* was present in all of the isolates. Vallet et al., [16], showed that *cupA* gene cluster plays a significant role in biofilm formation. They also indicated that *CupA*-dependent adhesions are more essential during the early stages of biofilm formation than type IV pili.

In our study, *pslA* gene was identified in 92% of the isolates. In a study, Emami et al., [23], showed that none of the negative biofilm samples contained the *pslA* gene, while 42% of the biofilm-positive isolates had the *pslA* gene. Ma et al., [38], indicated that the Psl is a substantial biofilm component playing a critical role in the resistance of *P. aeruginosa *species*.*

In conclusion, this study illustrated that the majority of clinical isolates of *P. aeruginosa* produced weak biofilm *in vitro*. It was also shown that the QS genes and virulence genes (*pslA* and *cupA*) were prevalent among the isolates. Identification of these genes and their association with biofilm formation can be advantageous in adopting therapeutic methods against *P. aeruginosa *infections.

## Conflict of Interest:

The authors declare no conflict of interest. 
